# Polymeric Membranes for Liquid Separation: Innovations in Materials, Fabrication, and Industrial Applications

**DOI:** 10.3390/polym16233240

**Published:** 2024-11-22

**Authors:** Lalit Ranjan Sahu, Diksha Yadav, Debasish Borah, Anuranjit Gogoi, Subrata Goswami, Gauri Hazarika, Sachin Karki, Moucham Borpatra Gohain, Saurabh V. Sawake, Sumit V. Jadhav, Soumya Chatterjee, Pravin G. Ingole

**Affiliations:** 1 Chemical Engineering Group, Engineering Sciences and Technology Division, CSIR-NorthEast Institute of Science and Technology, Jorhat 785006, Assam, India; lalitranjan00@gmail.com (L.R.S.); diksyadav96233@gmail.com (D.Y.); debasishbora051@gmail.com (D.B.); anuranjit@outlook.com (A.G.); subratagoswami098@gmail.com (S.G.); gauripriyahazarika98@gmail.com (G.H.); sachinkarkissk@gmail.com (S.K.); moucham1234@gmail.com (M.B.G.); saurabhsawake27013@gmail.com (S.V.S.); 2 Academy of Scientific and Innovative Research (AcSIR), Ghaziabad 201002, Uttar Pradesh, India; 3 Government Polytechnic, Hol Tarfe Haveli, Nandurbar 425412, Maharashtra, India; svjadhav84@gmail.com; 4 Defence Research Laboratory, Defence Research and Development Organisation (DRDO), Tezpur 784001, Assam, India; drlsoumya@gmail.com

**Keywords:** polymeric membranes, liquid separation, material innovations, performance enhancement, scalability and commercialization, fouling mitigation

## Abstract

Polymeric membranes have emerged as a versatile and efficient liquid separation technology, addressing the growing demand for sustainable, high-performance separation processes in various industrial sectors. This review offers an in-depth analysis of recent developments in polymeric membrane technology, focusing on materials’ advancements, innovative fabrication methods, and strategies for improving performance. We discuss the underlying principles of membrane separation, selecting suitable polymers, and integrating novel materials, such as mixed-matrix and composite membranes, to enhance selectivity, permeability, and antifouling properties. The article also highlights the challenges and limitations associated with polymeric membranes, including stability, fouling, and scalability, and explores potential solutions to overcome these obstacles. This review aims to guide the development of next-generation polymeric membranes for efficient and sustainable liquid separation by offering a detailed analysis of current research and future directions.

## 1. Introduction

Over recent decades, the membrane-based separation technology has increasingly captivated interest from researchers and industries globally, owing to its broad applications across numerous sectors. In recent years, commercial membranes have become widely available for water treatment [1,2,3]. Polymeric membranes have become a cornerstone in separation technologies due to their tunable properties, adaptability, and cost-effectiveness [4]. These membranes, primarily composed of synthetic polymers, are designed to selectively separate specific components from liquid mixtures, making them invaluable in various industrial processes [5,6]. In recent decades, advances in polymer chemistry and membrane fabrication techniques have markedly improved polymeric membranes’ performance, durability, and application range. Their ability to be engineered for specific separation tasks has positioned them as essential tools in fields ranging from water treatment to food processing and chemical manufacturing [7,8,9]. Polymeric membranes are a sustainable solution for industrial applications due to their adaptive chemistry, structure, and performance attributes. Enhanced separation efficiency and the creation of advanced structures through innovative polymer chemistry have resulted in membranes with optimized pore sizes and free volumes [10]. With the rapid growth of modern industry, agriculture, and the global population, it has become evident that many countries and regions are facing water scarcity and severe water pollution [11,12,13]. With the increasing global demand for clean water and more sustainable industrial operations, the urgency for developing cutting-edge separation technologies has reached an all-time high.

Today, membrane engineering is a dynamic scientific field focused on developing, applying, and optimizing various membrane technologies. This field enables the precise determination of the most suitable membrane operations for specific industrial processes [14,15,16,17]. The widespread adoption of membrane technologies across multiple industrial sectors has further reinforced their selection as the preferred solution [18]. Researchers have investigated various composite and nanocomposite membranes for water purification, demonstrating significant potential and versatility. Nour et al. studied mixed-matrix membranes (MMMs) incorporated by the metal-organic framework (MOF) for efficient nickel and cobalt removal. They highlighted the excellent nickel and cobalt capture efficiency of the resulting MOF@PES MMMs, even in the presence of common ions, signifying future research avenues for enhancing the functionality of MOF-based MMMs [19]. Integrating MOFs within polymer matrices is an advantageous strategy for improving their performance. MOFs are recognized as an efficient and promising technology for heavy metals’ removal from water due to several notable advantages, including microporosity, substantial water and structural stability, and the capacity for flexibility and adaptability, which are crucial for capturing and accommodating the targeted guest compounds [19,20,21,22]. 

In water purification, developing highly permeable antifouling membranes is a critical challenge for researchers, and significant progress has been made in this area [23]. Khoo et al. developed antifouling thin-film composite and nanocomposite membranes specifically designed with titania nanotubes (TNTs) [24]. More significantly, the produced TFN membrane demonstrated exceptional fouling resistance by obtaining a flux recovery rate of 85.77%, as opposed to the control membrane’s 57.94%. Incorporating inorganic nanofillers has also played a crucial role in enhancing the antifouling properties, making these membranes essential for providing purified water in a world where clean water access is increasingly vital [25,26,27].

This review provides an extensive overview of the most current developments in polymeric membrane technology for liquid separation applications. It explores various types of polymeric membranes, their fabrication methods, and the materials used in their construction, focusing on innovations that improve membrane performance. The review addresses the key challenges polymeric membranes face, such as stability, fouling, and scalability, and discusses potential strategies to overcome these obstacles. The scope of this review encompasses both established and emerging applications of polymeric membranes, offering insights into their commercialization potential and future research directions. This review aims to inform and inspire continued innovation in polymeric membranes for liquid separation by synthesizing current knowledge and highlighting cutting-edge developments.

## 2. Methodology

This review aims to comprehensively analyze polymeric membranes, highlighting advancements that address the critical need for high-performance, sustainable liquid separation in various industrial sectors, such as desalination and wastewater treatment. This review article serves as a resource for understanding the recent innovations in materials, fabrication techniques, and functional enhancements to maximize polymeric membranes’ separation efficiency and durability. This review encompasses the evolution of polymeric membrane materials and processes, with a detailed examination of advancements, such as phase inversion, electrospinning, interfacial polymerization, layer-by-layer assembly, and chemical vapor deposition (CVD). Emphasis is given to incorporating novel materials, such as nanomaterials and aquaporin, to improve membrane properties, such as the polymeric membranes’ selectivity, permeability, and antifouling abilities. In addition, it explores strategies to enhance membrane performance, such as chemical and plasma treatment techniques, and investigates the roles of mixed-matrix, composite, and nanocomposite membranes. The primary function of this review is to guide readers in understanding the scientific principles of membrane separation, such as size exclusion, Donnan ion exclusion, hydrophobic–hydrophilic interactions, and electrostatic interactions. The review offers insights into advanced membrane functionality and performance in real-world applications by examining the intersection of polymer science with nanotechnology and surface engineering.

Moreover, it provides a critical perspective on the limitations of polymeric membranes, including chemical and thermal stability challenges and fouling. It presents potential approaches to overcome these issues through emerging innovations in membrane separation technologies. Ultimately, the intent is to inspire and inform the development of next-generation polymeric membranes that meet the rising demands for sustainable water treatment and industrial applications. This review supports the advancement of polymeric membranes by providing an extensive knowledge base that inspires new research pathways and practical applications for mitigating water pollution and enhancing industrial sustainability using polymeric membranes.

## 3. Fundamentals of Polymeric Membrane Technology

### 3.1. History of Membrane Technology

**Figure 1:**
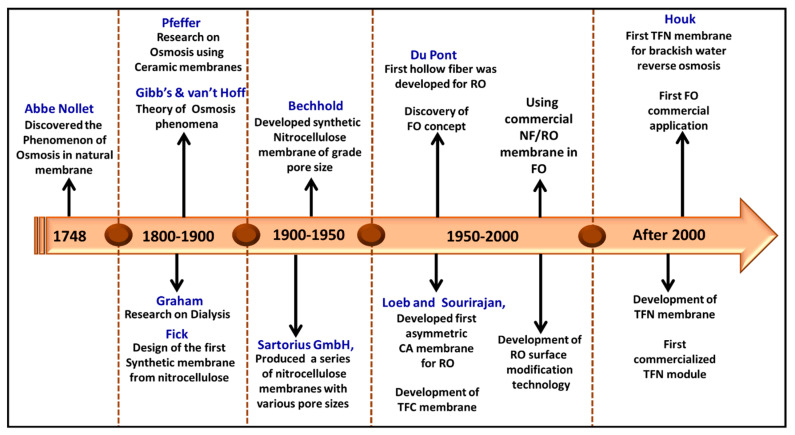
Significant advancements of membrane technology for liquid separation.

The history of membrane-based separation technology is rooted in extensive laboratory research that preceded its widespread industrial application. Initially, membranes were primarily a topic of scientific inquiry, with limited practical use. However, this changed dramatically in the 1950s, when the focus shifted to practical applications, leading to the rapid development of a significant membrane-based industry [28,29]. In the early 1950s, electrodialysis (ED), microfiltration (MF), and ion exchange membranes were employed and used on a lab scale in Europe to test drinking water safety [30]. However, polymeric membranes were first utilized for liquid material separation in the 1950s. Cellulose acetate, a cellulose derivative, became the primary material for developing polymeric membranes. In 1959, Sidney Loeb and Srinivasa Sourirajan developed the first practical polymeric membrane for seawater desalination at the University of California. They created an asymmetric membrane with a dense layer of cellulose acetate cast on a porous sub-layer for support. This membrane successfully turned saline water into potable water, leading to the construction of large-scale desalination plants to meet potable water demand [31,32]. With the addition of high-temperature thermoplastics, polymeric membranes became flexible, cost-effective, and applicable for various separations, including oil–water separation, protein separation, heavy metal ion separation, gas separation, dye separation, dialysis, and more. This versatility has led to a high demand for polymeric membranes in industries, such as dairy, fuel cells, packaging, petroleum, pharmaceuticals, sensors, textiles, medical, and energy sectors [33,34]. As a result, researchers have shown a deep interest in developing new polymeric membranes using various polymer materials for different purposes. Figure 1 illustrates the significant advancement of membrane technology.

With the advancements in polymer chemistry, many synthetic polymers were produced. Eventually, they became available to fabricate novel membranes with specific transport properties, flexibility, and exceptional thermal and mechanical stability. Based on the thermodynamics of irreversible processes, the transport properties of a membrane were illustrated by a comprehensive theory [31,35]. After the use of cellulose acetate polymers for membrane fabrication in the initial stage, soon, synthetic polymers, such as polysulfone (PSf), polyethersulfone (PES), polyamides (PA), polyacrylonitrile (PAN), polyethylene (PE), polyvinyl chloride (PVC), polyacrylonitrile (PAN), etc., were used as the primary material for the fabrication of synthetic membranes [31,36]. These synthetic polymers provide superior chemical and thermal stability and enhanced mechanical strength compared to cellulose acetate. Despite these advantages, cellulose acetate remained the dominant material for reverse osmosis (RO) membranes until the advent of interfacial-polymerized composite membranes [31,37].

### 3.2. Membrane Separation Process

**Figure 2:**
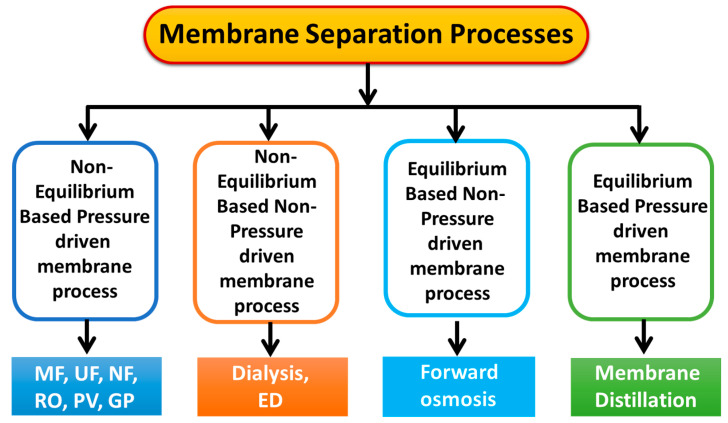
Different kinds of membrane separation processes.

Membrane separation primarily relies on three key principles: molecular sieving, adsorption, and electrostatic interactions. The adsorption mechanism in membrane separation primarily relies on the hydrophobic interactions between the solute (analyte) and the membrane. This kind of membrane results in a higher rejection rate due to the smaller pore size of the membranes [38,39]. This indicates that the separation primarily depends on the size of the pore and solute. This advancement spurred the development of a range of membrane separation techniques, including ultrafiltration (UF), microfiltration (MF), nanofiltration (NF), forward osmosis (FO), reverse osmosis (RO), gas separation, dialysis, electrodialysis, pervaporation (PV), membrane contactors, and membrane reactors [38,39,40]. Figure 2 illustrates a schematic overview of various membrane separation processes categorized by their driving forces. Due to the low energy requirement compared to conventional thermal separation technology, membrane separation is often considered cost-effective and environmentally friendly [41]. 

Unlike many other separation methods governed by phase equilibrium relations, membrane separation techniques fundamentally rely on the relative rates of mass transfer. Transport takes place through different mechanisms, such as solution diffusion for liquid separation. The rate at which each component permeates through a membrane depends on its capacity to dissolve and diffuse within the membrane material, which dictates the membrane’s selectivity. Transport through the membrane can be affected by factors such as pressure or temperature gradients, convection or diffusion driven by electric fields, or concentration differences [42]. 

### 3.3. Classification of Membrane Material and Structure

Membranes can be classified based on their structural morphology and the materials employed in their fabrication. Structurally, membranes fall into two main types: isotropic and anisotropic [43]. Isotropic membranes exhibit uniform properties throughout their structure and are composed of a single material type. They can be categorized as non-porous dense films, macro-porous, and electrically charged membranes. Macro-porous membranes feature pores ranging from 0.1 to 5 µm, enabling separation based on pore size. Non-porous dense-film membranes facilitate separation through diffusion driven by pressure and electrical gradients. Electrically charged membranes, also known as ion exchange membranes, separate ions according to their charge density, affecting transport rates via ion concentration and charge [43,44,45].

In contrast, anisotropic membranes have varied structural and chemical properties and are classified into composite and phase separation membranes. Composite membranes are characterized by an asymmetric structure featuring a thin, highly cross-linked top selective layer (usually <1 µm) on a thicker microporous support. The properties of this top layer, including porosity, pore size, and thickness, control the transport rate of the feed mixture [43,46,47]. Phase separation membranes comprise multiple layers of the same material but differ in porosity, pore size, and thickness across layers.

**Figure 3:**
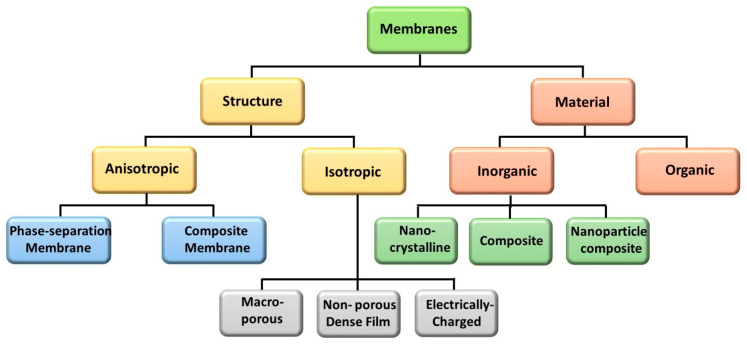
Classification of membranes based on structure and material.

Membranes are also categorized by the materials used in their fabrication, which can be inorganic or organic. Inorganic membranes are made from amorphous silica, carbon molecular sieves, graphene oxide, palladium alloys, zeolites, perovskites, etc. These membranes can be found in self-supporting structures or dense/porous multilayer supports. Polymers, such as cellulose acetate, polyimide, polysulfone, polyethersulfone, polycarbonate, polyvinylidene fluoride, and polydimethylsiloxane, are commonly used to fabricate organic membranes [43,48,49]. The membranes’ classification by their structure and material is illustrated in Figure 3.

## 4. Polymeric Membranes for Liquid Separation

### 4.1. Membrane Materials and Configurations

Membrane materials are either polymer or inorganic materials. The primary determinant in selecting materials for liquid separation membranes is the materials’ ability to separate. Inorganic materials, such as metals or ceramics, are also used to create membranes [50]. Inorganic ceramic membranes have been developed from the oxides of titanium, zirconium, aluminum, and silicon [51]. Ceramic membranes are frequently employed for UF and MF because they are chemically resistant, thermally stable, and microporous [50]. Because of their stability, ceramic microfiltration/ultrafiltration membranes are especially well suited for applications in the biotechnology, food, and pharmaceutical industries, where the membranes must be repeatedly cleaned with strong solutions and steam sterilized. Unfortunately, the disadvantages of mechanical brittleness and high expenses have hampered widespread application. Metallic membranes possess highly refined porosity, with stainless steel being a frequently used material for fabrication [52].

**Figure 4:**
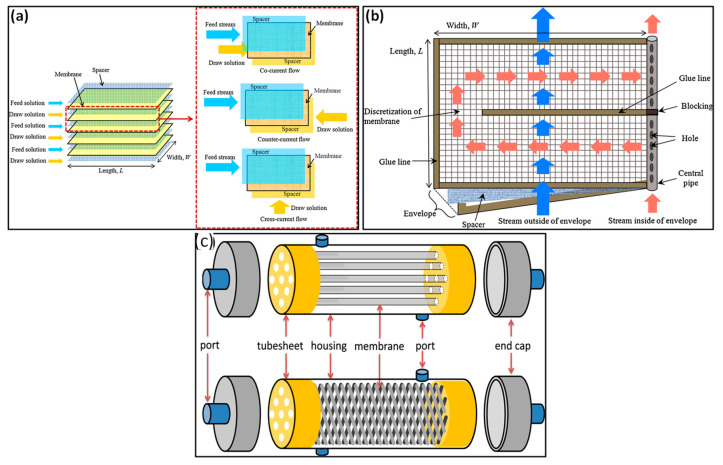
Schematic representation of planar, (**a**) plate-and-frame and (**b**) spiral wound [54], and cylindrical, (**c**) hollow-fiber membrane with parallel and crisscross arrangement [55], geometries.

Polymer-based membranes are widely used commercially due to their scalability and thermal durability. Synthetic organic polymers dominate the membrane market across UF, MF, NF, and RO filtration processes. RO membranes typically consist of a substrate material, such as polysulfone or cellulose acetate, and are then coated with a layer of aromatic polyamides [50]. Similarly, NF membranes are frequently constructed from blends of cellulose acetate or polyamide composites, although modified ultrafiltration (UF) membranes, such as sulfonated polysulfone, are also employed. These membranes can be assembled in different configurations, which refer to the arrangement and orientation of the membrane concerning the feed and permeate flow. The configurations are generally categorized into two primary geometries: planar (e.g., spiral wound and plate-and-frame) and cylindrical (e.g., hollow fiber and tubular) [53,54], as illustrated in Figure 4.

**Figure 5:**
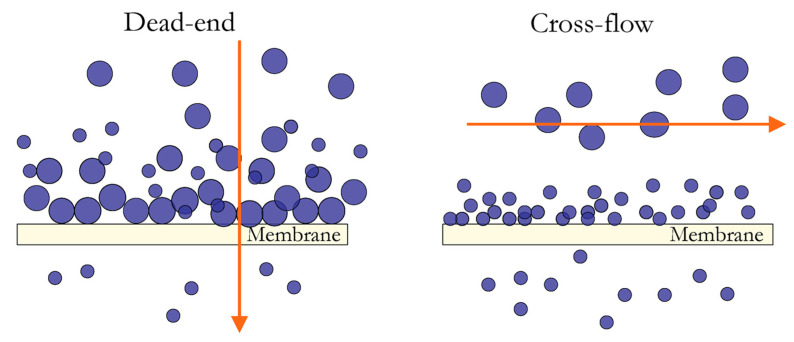
Representation of dead-end and crossflow filtration setups for membranes [55].

Planar geometry: The plate-and-frame or flat-sheet module is a fundamental membrane configuration featuring a flat-sheet membrane, spacers, and two end plates. The feed mixture flows across the membrane surface, passing part into a permeate channel collected in a central manifold. Due to pressure limitations, these modules are predominantly utilized for MF and UF and exhibit a relatively low surface-area-to-volume ratio. There are two types: dead-end, where the feed flows perpendicularly, and crossflow, where it flows tangentially, reducing membrane fouling and extending membrane life, as shown in Figure 5 [51,53,56].

A prevalent design in industrial applications for NF and RO involves flat-sheet membranes arranged in a spiral configuration around a perforated central tube. This setup allows the feed to flow along one side of the membrane while the permeate is collected on the opposite side, moving inward in a spiral pattern. This design is favored for its efficiency, due to its elevated surface-area-to-volume ratio [53].

Cylindrical geometry: Tubular modules consist of a membrane encased within a cylindrical tube, through which the feed is directed and pumped. They are primarily used for ultrafiltration due to their better fluid dynamics [51,54].

Hollow-fiber modules utilized in seawater desalination typically comprise bundles of hollow fibers encased within a pressure vessel. These modules may include a shell-side feed configuration, where the feed flows around the exterior of the fibers and exits from their ends. In a bore-side feed arrangement, hollow-fiber modules can circulate feed through the fibers [50,56]. It is not often the case that hollow fibers used in membrane bioreactors and wastewater treatment are also utilized in pressure vessels. The permeate is extracted from one end of the fiber bundles suspended in the feed solution. Hollow-fiber modules offer key benefits due to their compact design, which allows them to design thousands of square meters of area in one cubic meter of module volume [51,56]. While flat-sheet and tubular modules are still in use, they are increasingly replaced by hollow-fiber and spiral-wound module designs.

**Figure 6:**
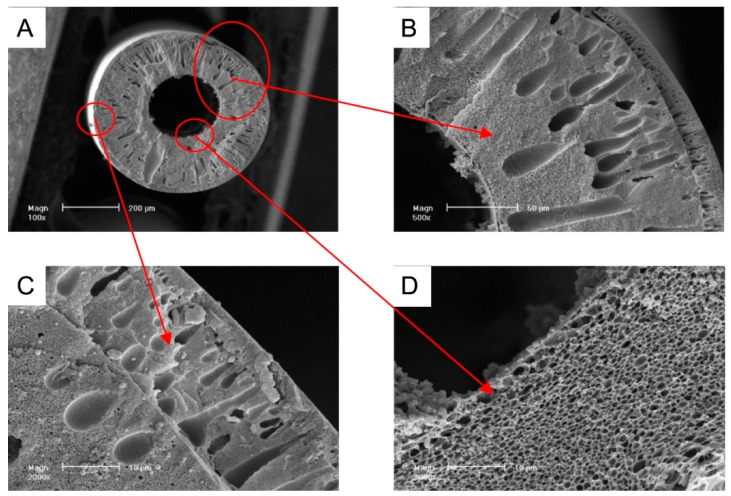
Cross-sectional SEM images of the dual-layer asymmetric hollow-fiber membrane [61]: (**A**) complete profile, (**B**) cross-sectional part, (**C**) outer layer, and (**D**) inner layer.

Fabrication of hollow-fiber membranes (HFMs) is more complicated than flat membranes. The fabrication of an asymmetric hollow-fiber membrane, whose SEM images are shown in Figure 6, consists of the following steps: dope preparation, degas, metering, spinning, evaporation, coagulation, and solvent exchange. The bore fluid (mostly pure water) and polymer dope are subjected to a spinneret. The extruded polymeric dope solutions from the spinneret can pass through an air gap before the coagulation bath. They are further transferred over rollers to collect the hollow fibers [57]. 

Choosing proper spinning techniques results in better HFM fabrication. The most commonly used spinning techniques for manufacturing HFMs include dry-jet wet spinning, melt spinning, wet spinning, and electrospinning. 

Wet spinning—Here, the air gap is absent; therefore, the extruded polymeric dope solutions from the spinneret are directly transferred to the coagulation bath containing non-solvent, where the dope solutions are precipitated and solidified. The just-spun fibers are collected over the roller, and before drying, a solvent exchange process is applied [58].

Dry spinning—Using air or inert gas in a warm air chamber, solid fibers are produced directly after extrusion from the spinneret by evaporating the solvent of the polymer dope solution, and further post-treatment is performed. 

Melt spinning—It uses melted polymer in the viscous form as the polymer dope solutions. The resulting hollow fiber is obtained by forcing the molten polymer within the spinneret, where quick cooling and solidifying are carried out.

Electrospinning—It is a modern technique to fabricate ultrafine fibers. Unlike traditional techniques, it operates by utilizing electrostatic forces with the help of a high-voltage electric field to stretch a polymer solution or melt it into thin fibers, without the need for high temperatures and solvents [59]. 

Dry-jet wet spinning—It has the advantages of both wet and melt spinning for HFM production [60].

### 4.2. Mechanism of Liquid Separation

Various mechanisms are involved depending on the characteristics of polymeric membranes, including factors such as surface charge, pore size, chemical properties, and the liquid being separated. However, while size exclusion is the main separation mechanism, other mechanisms exist, such as electrostatic interaction, hydrophobic–hydrophilic adsorption, affinity-based separation, ion exchange mechanism, etc. [62].

Size exclusion (sieving/steric exclusion): In the size exclusion mechanism, the separation of molecules or particles is determined by their size relative to the membrane’s pore size. Particles larger than the pores are retained on the membrane’s surface, while smaller particles pass through. Membranes are characterized by their molecular weight cut-off (MWCO), which exhibits their cut-off value—the molecules of lower molecular weight than MWCO of the membrane will not be separated by size exclusion. Size exclusion works predominantly for microfiltration (MF) and ultrafiltration (UF) [63].

Certain ions in aqueous solutions form hydration shells—clusters of water molecules surrounding the ions. Although these ions carry charge, their hydrated form becomes too large to pass through the pores of a membrane. This separation process is known as the dielectric exclusion mechanism, a subtype of size exclusion [64,65]. 

Electrostatic interaction (Donnan ion exclusion): In this mechanism, charged particles are separated based on their charge when interacting with a charged membrane surface. Charged membranes either attract or repel ions based on their charge. They attract counter ions and repel co-ions. A positively charged membrane (cationic membrane) repels positively charged ions and attracts negatively charged ions, while a negatively charged membrane (anionic membrane) repels negatively charged ions and attracts positively charged ions. The Donnan ion effect, which is the electrostatic interaction, increases with the charge of co-ions and decreases with the charge of counter ions. This mechanism is found in NF membranes, where the separation of monovalent and divalent ions is required. It is also used in membrane bioreactors and certain types of electrodialysis [64,66].

**Figure 7:**
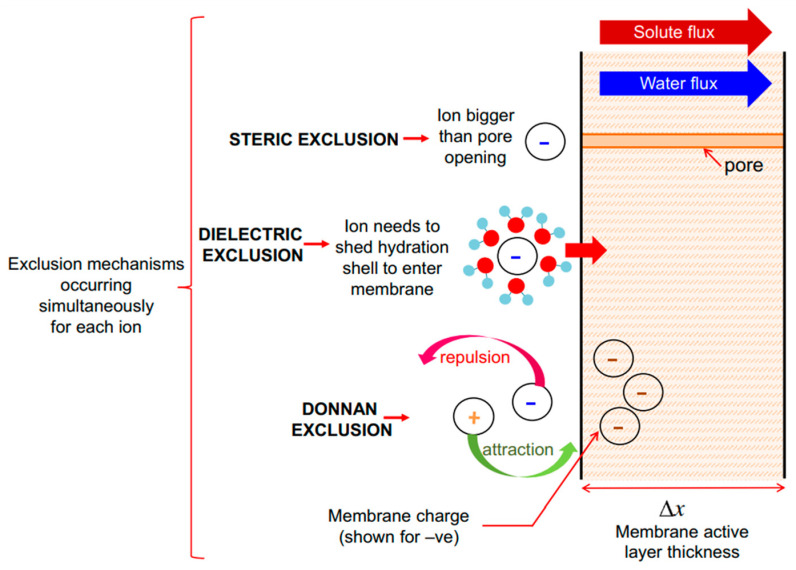
Diagram representing the size exclusion (steric exclusion), Donnan ion exclusion (electrostatic interaction), and dielectric exclusion mechanisms [65].

A comprised representation of the size exclusion, Donnan ion exclusion, and dielectric exclusion mechanisms is represented in Figure 7.

**Figure 8:**
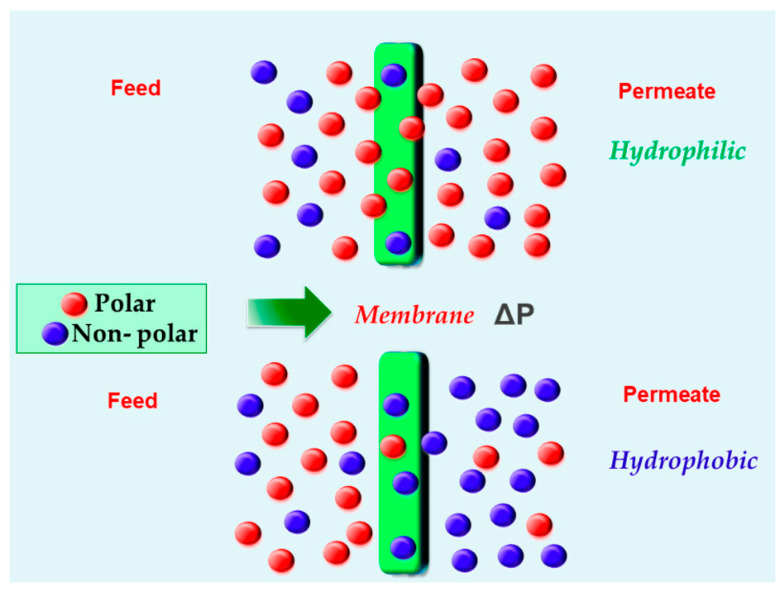
Schematic representation of hydrophilic and hydrophobic mechanisms (hydrophobic–hydrophilic interaction) of transportation [68].

Hydrophobic–hydrophilic interaction: Hydrophilic and hydrophobic membranes interact based on their chemical affinity. Due to their non-polar nature, hydrophobic membranes repel water but attract and allow organic solvents and non-polar compounds, as represented in Figure 8. On the other hand, hydrophilic membranes attract water and repel non-polar molecules. This mechanism is commonly observed in pervaporation membranes, where water molecules can pass through for organic solvent recovery. It is also relevant in oil–water separation processes. This mechanism also operates in membrane distillation, a thermally driven process where a hydrophobic membrane separates the vapor of a liquid from a mixture of liquids [67,68].

**Figure 9:**
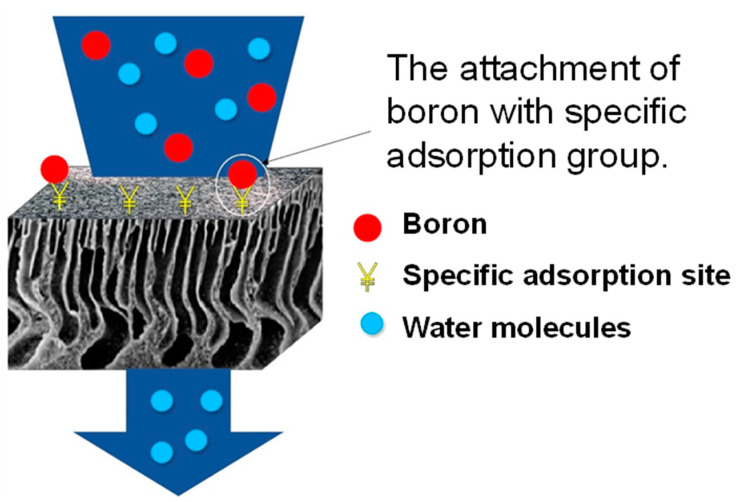
An illustration of capturing boron using an affinity-based adsorptive membrane [69].

Affinity-based separation (adsorption): In this type of separation, specific groups or ligands are attached to the surface of the membranes to act as receptors for certain types of molecules. This selective separation membrane is used to capture and collect valuable molecules, making it useful for obtaining an enantiomeric excess of one enantiomer from a racemic mixture and separating chiral molecules. Affinity membranes are mainly used in biotechnology and bioprocessing, especially for purifying proteins or antibodies. They are also important for sensor applications and medical diagnostics to capture specific biomolecules. Figure 9 shows how the membrane is utilized to capture boron, specifically by tailoring specific adsorption sites on the membrane surface [69,70].

**Figure 10:**
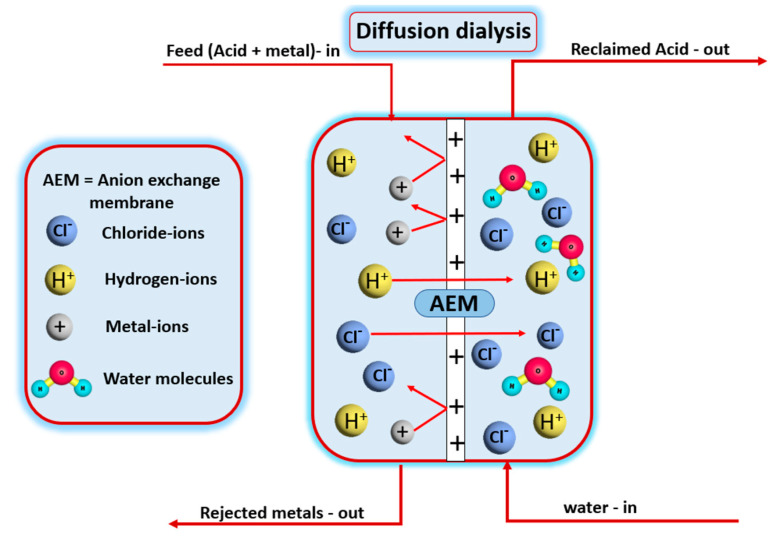
Diffusion dialysis using an anion exchange membrane (AEM) [73].

Ion exchange mechanism: In the ion exchange mechanism, ions are exchanged between a solution and the membrane. The membranes are either anion exchange or cation exchange membranes. For example, a cation exchange membrane has a negative charge on the surface. When a solution passes through, positive ions are attracted to the membrane, allowing it to pass but repel the negative ions. The ion exchange membrane differs slightly from the ion exchange resin, as a counter ion on the resin surface replaces the charged ion. This advanced mechanism is used for demineralizing and softening water, among other applications. Diffusion dialysis is another important application. As represented in Figure 10, an anion exchange membrane (AEM) is used to collect hydrochloric acid and reject metal ions. This can apply to recycling and reusing acids while producing chemicals and collecting metal ions as residual products [71,72,73].

### 4.3. Fabrication Techniques for Polymeric Membranes

Fabrication of polymeric membranes comprises an intricate multiscale process controlled by different aspects, such as the phase formation kinetics, thermodynamic potential differences, and mass transfer rates. The fabrication process of polymeric membranes can be better understood through three crucial scales. At the molecular scale, the polymer molecules interact with the solvent molecules and go to the nanometer (nm) and nanosecond (ns) length and time scales, respectively. Meanwhile, at the mesoscale, different ordered structures and phase domains with lengths in the range of 10–100 nm are achieved over a 10–1000 ns time scale. At the manufacturing scale, membranes are prepared in the micrometers (µm) range, with pore sizes ranging from 10 to 1000 nm, taking several seconds or even hours for fabrication [74,75,76,77,78,79]. Researchers have employed various innovative fabrication methods, some of which are detailed below, to strengthen and enhance polymeric membranes’ physical and chemical properties.

#### 4.3.1. Phase Inversion 

The most widely used method for producing microporous polymeric membranes is the phase inversion (PI) process, which was developed early in 1900. This method involves altering the physical state of a homogeneous polymer solution by coming in contact with other liquid/vapor phases or a temperature change, turning it into a solid phase. This process is used to create most polyamide membranes that are sold commercially [80,81,82]. Based on control parameters, phase inversion methods can be classified into four different types, namely, vapor-induced phase separation (VIPS), non-solvent-induced phase separation (NIPS) or immersion precipitation, thermally induced phase separation (TIPS), and evaporation-induced phase separation (EIPS) [83,84]. The idea behind the thermally induced phase separation (TIPS) method is that as the temperature drops, the solvent content usually decreases as well. Later, the solvent can be eliminated by freeze-drying, evaporation, and extraction after de-mixing [85]. The evaporation-induced phase separation (EIPS) method involves a solvent or a combination of volatile non-solvents to form the polymer solution. The solvent is removed through evaporation, forming a precipitate or de-mixing/precipitation. Solution casting is another name for this technique. 

Vapor-induced phase separation (VIPS) is a technique wherein a polymer solution is exposed to an environment containing a non-solvent, often water. De-mixing or precipitation takes place once the non-solvent is absorbed. The polymer solution is immersed in a coagulation bath containing a non-solvent (usually water) in a non-solvent-induced phase separation (NIPS) or immersion precipitation process. Mixing and precipitation occur as the polymer solution solvents trade places with the non-solvent coagulation bath. The solvent and non-solvent must be miscible for this to occur [86]. Among these processes, the NIPS method is mostly used for fabricating polymeric membranes, which involve liquid–liquid de-mixing and the diffusion rate of solvent–non-solvent as the deciding factors for the membrane’s overall structure and anatomy, such as a dense, spongy, porous, symmetric, and asymmetric nature [87]. The solvent and non-solvent used in this process diffuse across the interface, causing phase inversion, followed by the polymer solution’s solidification (precipitation). Several conditions, such as humidity, temperature, coagulation bath composition, and solvent properties, must be checked during this process [83]. In a recent study conducted by Fareed et al. [88], they developed high-performance pervaporation membranes using an innovative coactive delayed phase inversion method. This approach involved the co-hydrolysis of a polyacrylonitrile (PAN) support membrane. Specifically, they substituted the conventional non-solvent, i.e., pure water, in the coagulation bath with a 1.0 M NaOH solution while sustaining a temperature of 50 °C. This modification facilitated a simultaneous, delayed phase inversion process and hydrolysis. Ren et al. [89] developed a functionalized ultrafiltration (UF) membrane by modifying the casting solution and coagulation bath by adding a radical initiator and functional monomers. The team illustrated the effectiveness of the UF membrane prepared by incorporating radical polymerization and a non-solvent-induced phase separation strategy using zwitterionic sulfobetaine methacrylate (SBMA) as a functional monomer. 

#### 4.3.2. Electrospinning

**Figure 11:**
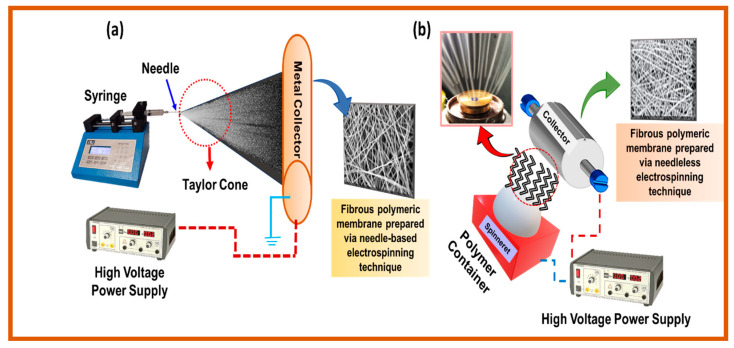
Diagram representing the electrospinning process for fabricating a fibrous polymeric membrane via the (**a**) needle-based electrospinning technique and (**b**) needleless electrospinning technique.

The electrospinning process is new and is used to create porous membranes for filtration and desalination, among other applications. A strong potential is applied between the grounded collector and the droplet of polymer solution. When the electrostatic potential is sufficiently increased, a charged liquid jet will occur to overcome the droplet’s surface tension. These fibrous membranes are special because the aspect ratios, i.e., length or diameter of the fibers and nano/microfibers’ shape, may be manipulated by adjusting the electric voltage applied, changing the flow speed and viscosity of the solution, as well as the environment in which the membrane is formed [90,91]. The ability to accurately manipulate the fiber size, morphology, and form has made electrospun fibrous membranes a preferred option for membrane filtration and membrane distillation (MD) applications. Research has shown that factors, such as the applied potential strength, polymer solution viscosity, ionic salt concentration, and solution feed rate, can significantly influence the nanostructure morphology and fiber diameter [92]. The four major components of an electrospinning system are typically a jet syringe, metal collector, spinneret (a fiber generator), and a high-voltage direct current (DC) power source (often 10–30 kV). There are two general categories for electrospinning: needle-based and needleless, as shown in Figure 11, based on the type of spinneret employed [93]. 

The viscous polymer solution is extruded from the syringe into the electric field while spinning through a spinning liquid thruster using a needle-based electrospinning method. This process charges the solution and, as a result, creates a repulsive interaction that counteracts the surface tension. The droplet changes into a jet after progressively taking the shape of a cone, called the Taylor cone. An unstable bending phenomenon is produced by the expelled jet. The polymer chain’s entanglement prevents the charged jet from breaking as it approaches the low potential zone while evaporating the solvent. At the other end, the metal collector collects the nanofiber [94].

On the other hand, nanofibers can be rapidly and directly electrospun from an open fluid surface using a process known as needleless electrospinning. This process, often associated with needle electrospinning, shapes multiple planes concurrently from the spinneret without any effect of the capillary action. Here, the flight starts a self-assembly phenomenon on the fluid’s free surface, making it difficult to regulate the spinning process in needleless electrospinning. This technique introduces a variety of spinneret shapes with varying production levels [95].

#### 4.3.3. Interfacial Polymerization

**Figure 12:**
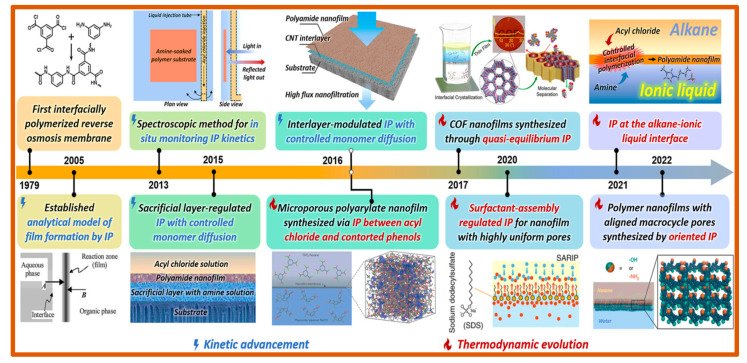
Significant advancements in the kinetic and thermodynamic aspects of polymer membranes created via liquid–liquid interfacial polymerization (IP) up to 2022 [100].

The widely accepted polymerization technique, interfacial polymerization, has been used to make composite membranes based on polyamides since 1979, as shown in Figure 12. Interfacial polymerization is a process driven by reaction-diffusion that primarily occurs at the interface between two immiscible liquids containing multifunctional monomers, typically following the Schotten–Baumann reaction [96]. This method enables precise control in fabricating fibers, polymer films, and capsules, making it highly valuable for various applications. The polymerization typically occurs at the interface between the aqueous-phase monomer (activator) and the organic-phase monomer (inhibitor), with the organic-phase monomer immiscible in the aqueous phase. Consequently, the reaction is localized at the interface, where a thin polymer film rapidly forms due to fast polymerization. The presence of catalysts or initiators can accelerate the chemical reaction. However, as the polymer film develops, it creates a barrier between the two phases, restricting the diffusion of monomers within the organic phase.

Consequently, the reaction rate decreases over time and eventually halts as the film thickens. This is because the polymer thin film thickens or the diffusion barrier increases. This indicates that the interfacial polymerization-produced film thickness is self-limiting since it increases rapidly initially and then gradually slows down to achieve a maximum over time [97]. The interfacial polymerization (IP) reaction involves the reaction of amine monomers, such as piperazine (PIP; used for creating a semi-aromatic polyamide structure) and m-phenylenediamine (MPD; for a fully aromatic polyamide layer), with acyl chlorides, such as trimesoyl chloride (TMC), occurring at the interface of aqueous–organic phase on the surface of porous substrates, such as polyethersulfone (PES), polysulfone (PSf), and polyacrylonitrile (PAN). This reaction forms the active layer of thin-film composite membranes [98]. As a diffusion-limited process, the IP reaction results in a denser and thinner active layer when highly reactive monomers are used, compared to less reactive ones. M-phenylenediamine (MPD) and trimesoyl chloride (TMC) are the most commonly employed monomers for developing functional polyamide layers in nanofiltration and reverse osmosis membranes [99]. The thickness and morphology of polymer films formed during polymerization are influenced by several factors, including the monomers’ solubility and diffusivity, reactivity, stoichiometry, concentration, morphology, temperature, additives, solvent media, etc. By controlling these features, one can create high-performance ultrathin polyamide membranes [100,101].

#### 4.3.4. Recent Developments in Fabrication Methods

Polymeric membranes are essential for numerous uses, such as emerging organic micropollutant removal, purification of water, and biomedical applications. Recent advancements in fabrication techniques have aimed to improve membranes’ mechanical strength, selectivity, permeability, and fouling resistance. In addition to the above-discussed fabrication techniques, many fresh and innovative processes are presently employed to manufacture advanced polymeric membranes, such as grafting, coating, layer-by-layer assembly, track-etching, incorporation of nanomaterials, the inkjet printing method, incorporation of aquaporin, as well as utilization of glassy polymers [102]. Numerous methods for producing polymeric membranes have been documented in the literature. For instance, since the early 1990s, diamond-like carbon (DLC) membranes have been fabricated using the chemical vapor deposition (CVD) technique [103]. Ultrafiltration freestanding DLC membranes were developed in 2012 using a parallel-plate plasma-enhanced CVD reactor [104]. The membranes exhibit excellent stability and remarkable mechanical strength in organic solvents, attributed to sp3 carbon networks within their structure. Thus, the CVD process offers a practical way to create membranes that may be adjusted in thickness. By fine-tuning the membrane properties for ionic and molecular separation applications, future advancements may enable the development of high-performance ultrathin membranes through the layer-by-layer (LBL) CVD technique [97].

##### Surface Modifications and Functionalization

**Figure 13:**
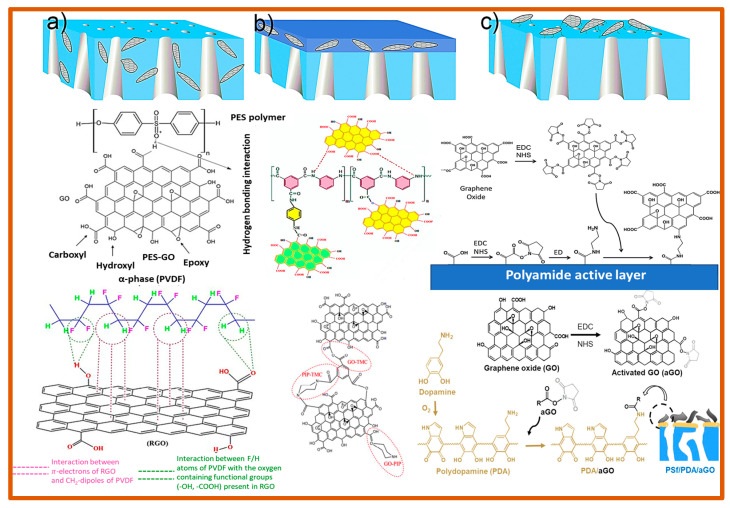
(**a**) Diagram showing the interaction of embedded carbon nanofillers with PES and PVDF polymers in UF membrane during the phase inversion process. (**b**) Interfacial polymerization process in NF and RO membranes between amine and TMC using carbon quantum dots (CQDs) and GO nanomaterials. (**c**) The surface modification of the membranes utilizing carbon nanofillers through covalent cross-linking [110].

To enhance membrane performance, especially concerning chemical stability, permeability, selectivity, and fouling resistance, modifying the surface morphology and functionalizing the membrane surface are essential approaches. By manipulating the membrane surface, these procedures can add new features or properties without affecting the overall properties of the membrane material. Researchers frequently utilize different techniques to modify the polymeric membrane’s surface, which improves the surface physicochemical properties of the cross-linked polyamide networks and overcomes their limits, as shown in Figure 13. Adding nanomaterials, for instance, silver, silica, titanium dioxide (TiO_2_), nickel oxide (NiO), aluminum oxide (Al_2_O_3_), iron oxide, GO, and CNTs, during the fabrication leads to nanocomposite polymeric membranes, significantly enhancing their properties [105,106]. Metal-based nanoparticles have been widely researched for their potential in water purification applications because of their remarkable adaptable characteristics, including high surface area, good selectivity, antifouling and antimicrobial properties, and increased hydrophilicity. Using metal complexations and coatings, a suitable surface modification can improve their adsorption affinity, selectivity, and reactivity even more [105]. Membrane surfaces can be functionalized with graphene oxide and carbon nanotubes to improve their mechanical strength, chemical resistance, and antifouling qualities. By creating targeted channels for water, these materials can also increase selectivity and permeability [106].

Moreover, plasma treatment techniques can introduce functional groups that strengthen chemical resistance or antifouling qualities. The plasma treatment activates the membrane surface, and monomers or polymers are grafted [106]. To create a multilayer structure, the layer-by-layer (LBL) method entails the alternating deposition of nanoparticles or polyelectrolytes that are oppositely charged on the membrane surface. With this method, one may precisely regulate the membrane’s permeability, pore size, and surface charge [107]. The coating is another significant method to modify the surface of the polymeric membranes, which includes photocatalytic coating: applying a layer of photocatalysts, such as titanium dioxide (TiO_2_), to membranes allows them to have self-cleaning capabilities when exposed to light, which lowers fouling and preserves the performance as time passes; hydrophilic coating: protein adsorption and biofouling can be minimized by coating the membrane surface with hydrophilic substances, such as polyethylene glycol (PEG) or zwitterionic polymers; antimicrobial coating: to stop the bacterial growth and biofilm formation, membranes can be functionalized with antimicrobial substances, for instance, silver nanoparticles, chitosan or quaternary ammonium compounds; dip coating: cross-linking agents (aqueous-phase and organic-phase monomers) are utilized to form covalent links between polymer chains to improve the thermal stability, mechanical strength, and chemical resistance of the membrane surface [108,109,110,111,112]. Chemical treatment is another widely recognized technique for modifying the surface characteristics of polymeric membranes by introducing different functional groups, such as amine, carboxyl, sulfone, and hydroxyl groups. Chemical treatment primarily involves hydrolysis, substitution, addition, and oxidation. When applied directly to the polymer solution, bulk modification techniques are straightforward and efficient. The backbone of aromatic polymers is frequently modified in bulk to increase surface hydrophilicity via amination, sulfonation, carboxylation, and epoxidation [108]. 

##### Mixed-Matrix Membranes (MMM)

**Figure 14:**
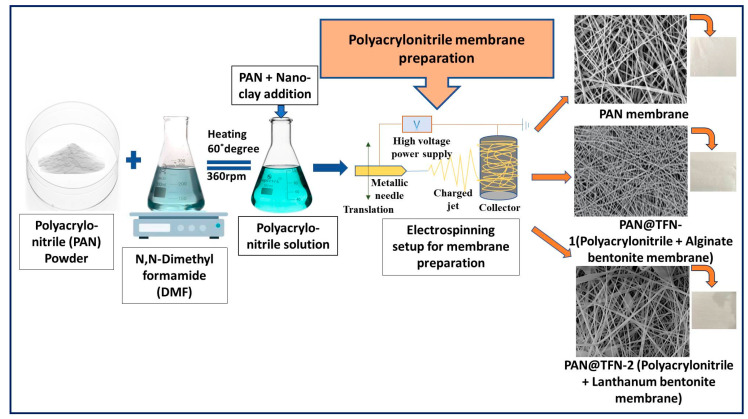
Electrospun nanofibrous MMMs’ development via the electrospinning technique [90].

Recently, researchers studying membranes have been experimenting with incorporating nanoparticles into traditional polymeric membranes. Significant advancements have been made in the last few years in creating nanomaterial-based polymeric nanofiltration membranes. These membranes demonstrate superior mechanical and thermal stability and effective antifouling characteristics. They also promise to address the traditional “trade-off” between solute selectivity and solvent permeability. The criteria that should be satisfied to fabricate sophisticated nanomaterial-based nanofiltration membranes are good nanomaterial compatibility with polymers, well-dispersed nanomaterials, and robust and stable nanomaterial–polymeric matrix interactions [113]. When nanofillers are incorporated into monomer/polymer solutions before membrane fabrication, the resultant nanofiller-blended membranes are known as mixed-matrix membranes (MMMs). Mixed-matrix membranes combine the advantages of inorganic materials’ mechanical strength and functional qualities with the low cost and convenience of producing membranes. MMMs aim to leverage the mechanical robustness and functional qualities of inorganic materials while benefiting from the cost-effectiveness and ease of membrane fabrication typically associated with polymeric membranes. To overcome the shortcomings of polymeric membranes for gas separations, Zimmerman and his coworkers initially presented mixed-matrix membranes in the 1990s [114]. MMMs containing inorganic molecular sieves, such as silicalite and zeolites, are incorporated into the polymer matrix to give the target species preferential flow routes [115,116]. Figure 14 depicts the synthesis of a mixed-matrix membrane prepared using the electrospun technique for liquid separation [90]. The “percolation threshold”, a volume fraction of filler material, is thought to be the point at which continuous paths of fast diffusion molecular sieves begin to form. Target molecules can now pass through the filler and the whole membrane cross-section [117,118]. Defects typically arise at the polymer–filler contact at a particularly high-volume fraction, reducing the selectivity. Due to their higher selectivity, targeted functions, and enhanced chemical, mechanical, and thermal stability, MMMs also provide the possibility of adjustable water treatment membranes.

##### Composite and Nanocomposite Polymeric Membranes

Achieving favorable outcomes without risking the membrane’s integrity requires balancing improved characteristics and defect formation [118]. A significant advancement in membrane separations was the fabrication of thin-film composite (TFC) membranes, which feature a porous polymeric support layer with an exceptionally thin “barrier” layer polymerized in situ. The terms “interfacial composite”, “composite”, or “TFC” membranes are frequently used to refer to these membranes generally, whereas layers incorporated with nanomaterials are referred to as thin-film nanocomposite (TFN) membranes. The primary advantage of TFC membranes is the ability to selectively tailor the chemistry of the upper selective layer and the porous support layer to enhance the overall functionality [118]. Phase inversion is typically used to create the porous layer, and IP or coating, such as dip, spray, or spin, is used to apply a cross-linked dense layer. The degree of polymer cross-linking is often increased by curing via heat, UV, and chemical treatment, which has a major effect on the thin film’s stability, permeability, and selectivity [119]. TFC membranes, known for their remarkable salt selectivity and elevated water permeability, have been effectively engineered using polyurea for water treatment applications [120]. The vapor phase IP technique is becoming popular and successful in creating polymer-based TFC membranes or covalent organic framework (COF) membranes with an ultrathin separation layer [121]. 

Using additives, such as metal-organic frameworks (MOFs), layered double hydroxide, clay minerals, and nanomaterials, improved the separation efficiency. Membranes prepared by the addition of nanomaterials are regarded as nanocomposite polymeric membranes. Numerous investigations have been conducted on the impact of various nanomaterial types on the composition and functionality of TFN membranes up to this point, as mentioned above. Recently, researchers have been interested in the topic of polymer/clay nanocomposites because of the relationship between the filler and polymer, the effect of the nanoscale structure, the easily adjustable pore size of nano-clay particles, the distinctive chemical composition, and the high adsorption capacity [122]. Clay is classified as a two-dimensional nanomaterial with an anisotropic sheet-like morphology, in contrast to the previously described metal-based nanofillers for TFN. Aside from its hydrophilic nature, the most distinctive property of clay is the positive or negative permanent charge, which results from the isomorphous substitution in the crystal structure of the clay layers. Dong et al. [123] developed TFN reverse osmosis (RO) membranes by embedding a cationic clay, montmorillonite (MMT), and an anionic clay, layered double hydroxide (LDH), having dimensions on a hundred-nanometer scale and a high aspect ratio. Because of these nano-clay filler materials’ special charged characteristics and hydrophilic nature, the properties of the TFN membranes were successfully altered. TFN membranes loaded with MMT and LDH demonstrated enhanced hydrophilicity, improved antifouling performance, and better desalination results. In another study by Selvan et al. [124], they utilized Kaolin clay and fabricated a zero-valent, iron-kaolinite, composite-based polyethersulfone (PES) membrane to separate As_2_O_3_ from water. The prepared nanocomposite membrane achieved a maximum removal efficiency of 50% for As_2_O_3_ from water. 

##### Biopolymer-Based Membranes

In many industrial domains, membrane technologies have become a viable substitute for energy-intensive separation procedures. The production of separating membranes poses a sustainability problem that calls for an important shift away from petrochemical-based raw materials and toward more environmentally friendly bio-based sources. Hardian et al. [125] worked on the development of oil- and solvent-resistant NF membranes using blends of chitosan (received from shrimp farming waste) and cellulose (plant-based material). The membrane’s molecular sieving capabilities were refined by varying the cellulose-to-chitosan ratio. Even at the highest temperature of 100 °C, the membranes demonstrated exceptional separation efficiency and chemical stability in harsh solvents, including polar aprotic solvents. The membranes demonstrated consistent separation performance after seven days of crossflow nanofiltration.

Jiang and his colleagues [126] employed the freeze-casting process and chemical vapor deposition to construct a novel superhydrophobic biopolymer aerogel membrane (T-SA/ligninx/rGO-MTMS) using graphene oxide (GO), sodium alginate (SA), and lignin. This innovative membrane significantly increased the oil removal efficiency, rising from 73.8% to 98.6%. Trimethoxymethylsilane (MTMS) undergoes silylation growth during production to provide a rough, hydrophobic surface for the ultimate protuberance. Even after 10 cycles of oil/water separation and oil sorption, T-SA/ligninx/rGO-MTMS showed promising cycling performance due to the ample protuberance and low-surface-energy reduced GO (rGO). 

To create a more sustainable solution for the membrane fabrication industry, Tomietto et al. [127] explored a novel bio-based material combination for the phase inversion process, which included a polyhydroxyalkanoate (PHA) as a biopolymer and Cyrene as a green solvent. Epoxidized broccoli vegetable oil (EBO), polyethylene glycol (PEG), and polyvinylpyrrolidone (PVP) were all effective pore-forming agents for these membranes. Several phase inversion process factors were examined to determine the various membrane microstructure options and their ultimate performances. The use of dense membranes in pervaporation (PV) has proven successful in separating organic/organic azeotropic mixtures.

## 5. Applications of Polymeric Membranes

**Figure 15:**
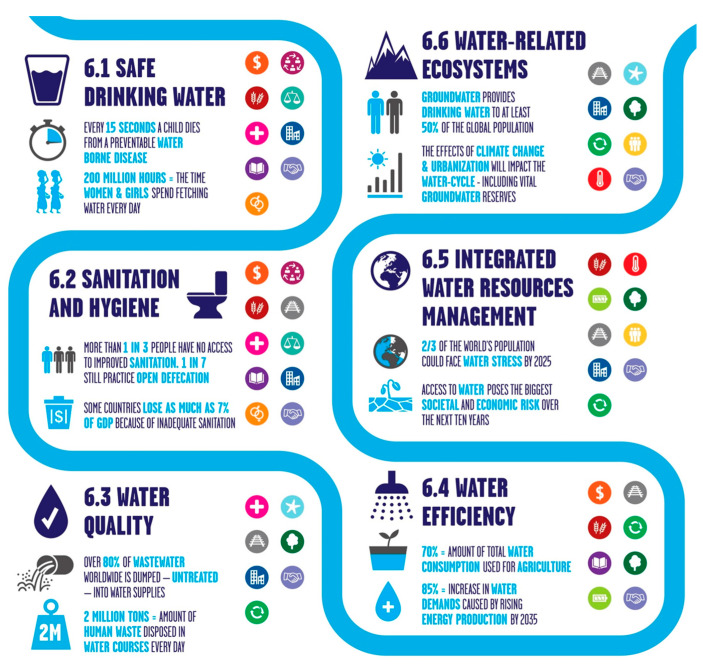
Diagrammatic representation of SDG 6 goals and its subgoals [131].

Polymeric membranes have numerous applications in various liquid separation areas, whether the requirement is purification or liquid separation. Desalination and cleaning brackish water were the initial applications; now, polymeric membranes are utilized in dairy, chemical, food, biotechnological, pharmaceutical, metal ion, beverages, metallurgy, dye, and other separation processes [128,129,130]. Developing polymeric membranes for water treatment and desalination is crucial for advancing global water sustainability, aligning with the United Nations Sustainable Development Goal 6. The SDG 6 and its subgoals are represented in Figure 15 [131].

### 5.1. Desalination and Dye Wastewater Treatment

Researchers have extensively investigated various types of polymeric membranes to achieve diverse requirements and applications. Jiang et al. used poly(m-phenylene isophthalamide) (PMIA) to prepare hollow-fiber membranes of the NF category via dry–wet phase inversion technology. They applied this membrane to the separation of Na_2_SO_4_ and MgSO_4_ and achieved ~98% and ~94% rejection from their 2000 ppm solution. They have also shown that the membrane can effectively separate Janus green B and Chromotrope 2B dyes [132]. Li et al. fabricated a thin-film flat-sheet membrane utilizing PMIA on porous epoxy support for dye and salt separation. Although they achieved >99% rejection of various dyes, their membranes poorly separated the metal salts [133]. 

In a study by Usha et al., a microfiltration membrane made from polypropylene (PP) was employed to separate oil/water emulsions. Their modified membrane exhibited super hydrophilicity and underwater oleophobicity, which is great for an energy-saving gravity-driven separation [134]. 

PVDF hollow-fiber membranes were modified by Sairiam and his group using pulse inductively coupled plasma (PICP) and grafted by chloro-alkyl-silanes to enhance hydrophobicity. They applied this membrane for the ozonation membrane contractor process to decolor the azo dye DB 71 (Direct Blue 71). They reported that their membrane decolored DB 71 in 90 min and removed 63% and 20% COD and TOC, respectively [135]. Borban et al. developed electrospun nanofiber mixed-matrix membranes (MMMs) by incorporating lanthanum-bentonite (La-Bnt) and alginate-bentonite (Alg-Bnt) into polyacrylonitrile (PAN). The electrospun membranes demonstrated impressive performance, with water permeability reaching 18.8 Lm^−2^·h^−1^ and rejection rates up to 99.08% for various micropollutants [90].

**Figure 16:**
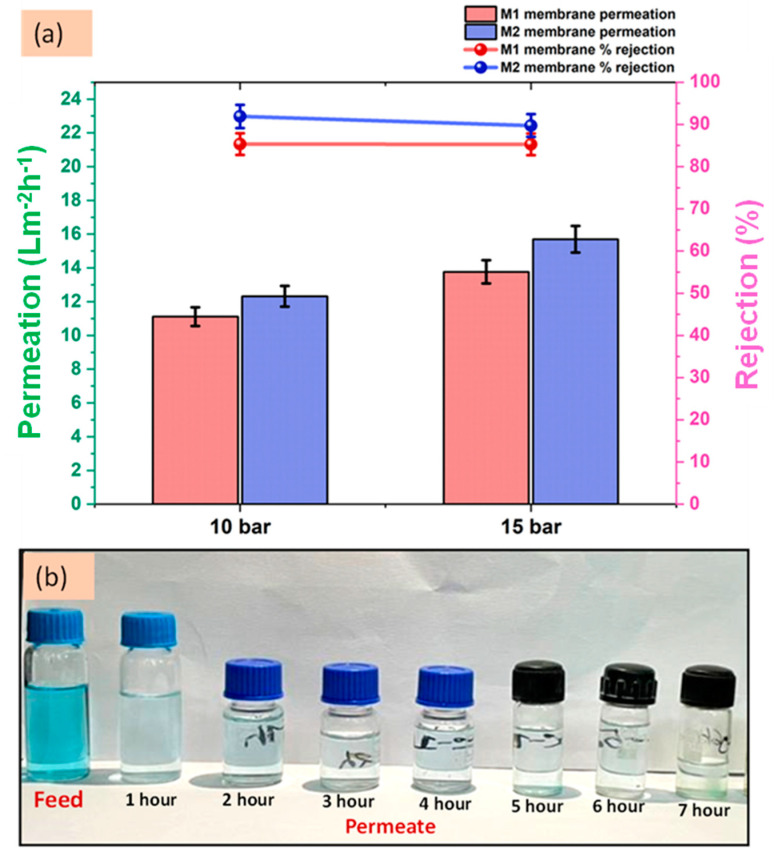
(**a**) Permeation and rejection graphs of M1 (without nanomaterial) and M2 (0.02 wt% UiO-66-NH_2_) nanofiltration membranes for malachite green dye at 10 and 15 bar. (**b**) Images to compare the dye feed and obtained permeate per hour for up to 7 h at room temperature [136].

In another study, Gohain et al. developed thin-film nanocomposite (TFN) membranes via interfacial polymerization (IP) between an aqueous phase containing piperazine (PIP) and UiO-66-NH_2_ and an organic phase containing trimethyl chloride (TMC) in n-hexane. These membranes were designed to separate phosphate and malachite green dye from water at low concentrations. The TFN membranes achieved excellent separation, with a rejection rate of 91.90% for malachite green (MG) and a flux of ~13.32 Lm^−2^·h^−1^. In comparison, phosphate exhibited a rejection rate of ~78.36% with a flux of ~22.22 Lm^−2^·h^−1^. The membranes also demonstrated a strong antifouling capability, with an antifouling tendency of ~84% and only ~14% irreversible fouling [136]. Figure 16a represents the permeation and rejection values obtained by their membranes (M1 and M2) against malachite green dye at 10 and 15 bar. To visually realize the performance, images of the permeate at each hour were taken, which can be seen in Figure 16b [136]. In another work, Gohain et al. developed MMMs by incorporating COOH-TiO_2_-functionalized GO nanoparticles with cellulose acetate extracted from discarded cigarette butts and studied for dyes and salts’ separation. The prepared membranes rejected up to 95% of dyes [137]. 

### 5.2. Metal Ion Removal from Wastewater

Nowadays, thin-film composite/nanocomposite and MMM types of polymeric membranes are prominently used for ion separation in the NF process. Due to their small size, metal ions are difficult to remove, and the Donnan ion effect (an electrostatic interaction mechanism) becomes more pronounced during their separation. In previous works, they successfully removed salt ions, especially heavy metal ions, which are required in industries for energy applications or removal in desalination [138,139].

**Figure 17:**
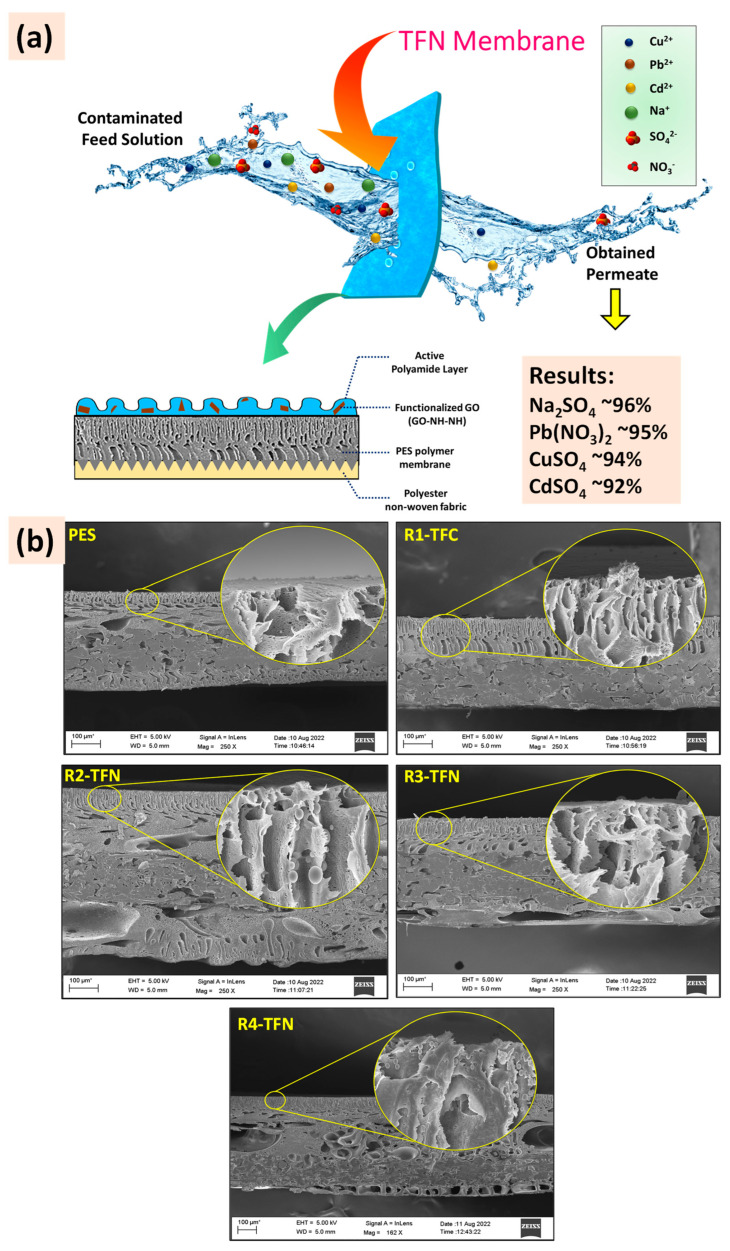
(**a**) TFN membranes prepared by the VP-IP method and (**b**) cross-sectional FE-SEM images of the prepared TFC, TFN, and pristine PES polymeric membranes [5].

Incorporating COOH-TiO_2_ nanofillers in the polyamide membrane prepared via the new vapor-phase interfacial polymerization (VP-IP) method on the polysulfone support provided rejection of Na_2_SO_4_, HgCl_2_, CuSO_4_, and Pb(NO_3_)_2_ salts up to ~87%, ~77% ~84%, and ~83% from their 2000 ppm contaminated aqueous solutions, respectively [140]. Using other functionalized nanomaterials, NHST and GO-NH-NH_2_, rich in amine groups, delivered cationic thin-film nanocomposite (TFN) membranes. These membranes were developed simply by the IP, and dip-coating using NHST (amine-rich synthetic talc) as nanofillers for polyamide TFN membranes exhibited ~99%, ~95%, and ~94% rejection of Na_2_SO_4_, MgSO_4_, and CuSO_4_ salts with pure water permeation up to 38 Lm^−2^·h^−1^·bar^−1^ [141]. TFN membranes were developed using another approach, integrating GO-NH-NH_2_ nanosheets as nanofillers and indulging in the spray coating method, as shown in Figure 17a. The nanosheet addition influenced the prepared membranes cross-linking density, as evidenced by the SEM images in Figure 17b. Membranes with a higher concentration of the nanosheets exhibited denser cross-linked polyamide surfaces. Prepared R4-TFN and R3-TFN membranes with higher nanofiller loading appeared comparatively denser. These prepared membranes exhibited pure water flux up to ~47 Lm^−2^·h^−1^ at 10 bar, and their separation quality was also good. They demonstrated ~96%, ~95%, ~94%, and ~92% rejection of Na_2_SO_4_, Pb(NO_3_)_2_, CuSO_4_, and CdSO_4_ salts, respectively [5]. 

Lithium-ion and polymer batteries are used for all kinds of electrical equipment, especially smartphones and laptops. Lithium extraction using membrane technology is far more efficient than other techniques, as articulated by Li et al. [142]. The applicability of NF membranes for the recovery of LiCl from seawater was first investigated by Wen et al. [143]. Zante prepared supported ion liquid membranes for extracting lithium from a complex aqueous solution. They displayed the separation of lithium from magnesium, cobalt, and nickel, and their lithium flux was 7 × 10^−7^ mol·cm^2^·s^−1^, with membrane stability up to two days [144]. Zhu et al. developed a composite membrane utilizing poly(vinylidene fluoride-co-hexafluoropropylene) (PVDF-HFP) as the host material reinforced with a nonwoven fabric. The prepared membrane exhibited an increased Na^+^ ion transference number and excellent electrolyte retention, maintaining its performance even at 110 °C [145].

### 5.3. Ion Removal and Recovery in Fertilizer Production

Nitrogen and phosphorus are key ingredients for fertilizer production to increase crop yield. A high quantity of ammonia is generated from pharmaceuticals and other waste found in water, which needs to be recovered to enhance nitrogen fertilizer production. Atsbha et al. [146] utilized commercial hollow-fiber membrane contactors (HFMC) to remove and recover ammonium from pharmaceutical wastewater at bench and pilot scales. The recovered ammoniacal nitrogen can be used to make ammonium-sulfate fertilizer. Composite membranes with active and support layers effectively recover ammonia during wastewater or potable water treatment. Shahgodari et al. [147] recovered ammonia from wastewater using a thin-film composite forward osmosis polymeric membrane by determining the permeability coefficient of NH_3_. They found a recovery of 15% of NH_3_ and, simultaneously, 35% of water at a pH of 11.5. A study by Kurama et al. [148] revealed that 96.9% of ammonium ions were recovered from potable water using RO membranes.

Phosphorus removal and recovery from wastewater has great potential in the fertilizer industry, particularly in producing phosphorus-based fertilizers through membrane processes. In a study by Kannan et al. [149], a high level of phosphorus was recovered in the form of calcium phosphate for making fertilizer using an anaerobic membrane bioreactor. Results obtained show variability in phosphorus removal due to the high carbonate alkalinity of the permeate, which was precipitated as calcium carbonate. This was overcome by aeration and the addition of acid, leading to 97% phosphorus removal. In a wastewater treatment study, Santos et al. [150] utilized two commercialized polyamide NF membranes, NF90 and NF270, to remove and recover phosphorus from surface water. They found that NF270 had a higher water flux than NF90; however, NF90 had a superior removal efficiency (>90%), with more than 96% of phosphorus recovered from surface water. In another study, Gerardo et al. [151] recovered nitrogen and phosphorus from dairy farm sludge using crossflow membrane filtration.

## 6. Challenges and Limitations

Despite notable advancements in polymeric membranes, some challenges still need to be addressed. One prominent issue is membrane fouling, which occurs when microorganisms and particles accumulate on the membrane surface. Researchers are extensively exploring innovative materials with enhanced performance and resistance to fouling. Furthermore, adapting these membranes for industrial applications presents a substantial challenge. Ensuring their durability and long-term stability under harsh operational conditions is crucial, affecting their effectiveness and feasibility in industrial environments [2]. Key challenges associated with membrane-based separation technologies include maintaining membrane stability, fouling, and mitigating degradation.

### 6.1. Chemical and Thermal Stability

Many studies have been conducted to improve the thermal properties of polymeric membranes and expand their applications. Various types of polymeric membranes have been investigated, including cellulose acetate (CA), PAN, PSf, PES, and poly(vinylidene fluoride) (PVDF), with a focus on advancing membranes for water and wastewater treatment. Table 1 shows some of the high-temperature-resistant polymers. Nevertheless, most polymeric membranes are not resilient to severe chemical and thermal environments. To ensure optimal performance of these membranes, heat exchanger units are often employed to lower the temperature of the inlet stream to below 50 °C, increasing operational costs and energy consumption [152].

Polymers with high thermal stability are typically insoluble in most common organic solvents. This resistance is attributed to their melting temperatures (Tm) and high glass transition (Tg) resulting from their rigid molecular structures and robust intermolecular forces. Although these properties contribute to their durability, they also pose difficulties in processing these polymers into various shapes, including films and fibers. Additionally, many thermally stable polymers are not biodegradable, have high environmental resistance, and can release harmful derivatives, posing environmental and human health risks [156]. To address these challenges, biodegradable and environmentally friendly polymers offer a promising alternative [150]. Biopolymers frequently exhibit lower mechanical and thermal stability relative to traditional polymers. Strategies to enhance their performance include blending biopolymers with more robust, stable polymers to improve mechanical strength and durability, cross-linking biopolymer chains to enhance chemical resistance and stability, and fabricating multilayer membranes with strong interlayer adhesion to optimize separation properties.

**Figure 18:**
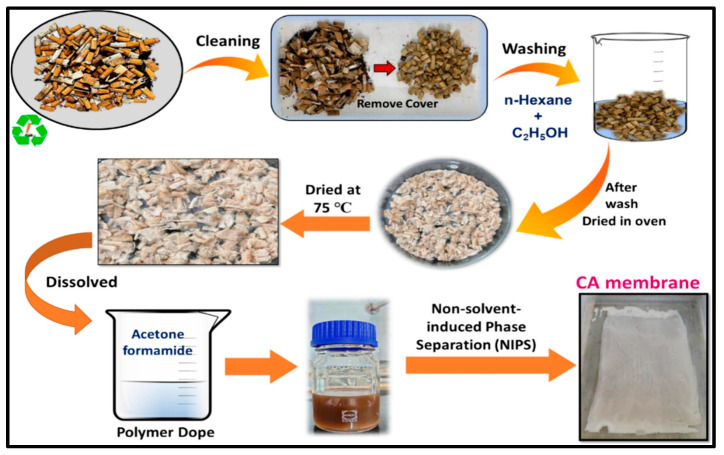
Cellulose acetate membrane fabrication using cigarette butt waste via the phase inversion process [137].

Additionally, incorporating nanomaterials into biopolymer matrices can significantly improve selectivity and permeability by introducing novel pathways and enhancing overall membrane performance [157,158]. Cellulose is a biopolymer consisting of repeating D-glucose units linked by β-1,4 glycosidic bonds. Its crystalline arrangement and tight chain packing provide superior chemical and thermal stability [159]. Figure 18 illustrates a cellulose-based membrane made by Gohain et al. [137], in which cellulose was taken out of cigarette butts to convert waste into usable products.

Integrating inorganic nanofillers into polymer matrices is a well-established approach for improving thermal stability and enhancing interfacial adhesion between inorganic and organic components, thereby improving the membrane performance [160,161,162]. Nanomaterials, such as silica (SiO_2_), GO, SiO_2_@GO, MOFs, salicylate-aluminoxane, zeolitic imidazolate frameworks (ZIFs), and metal oxides, are commonly employed in composite membranes [163,164,165,166,167,168,169,170,171,172]. These fillers restrict polymer chain mobility, improving thermal stability [173]. Khorshidi et al. developed high-performance RO nanocomposite membranes via in situ polymerization by integrating surface-modified TiO_2_ and oleic acid into a polyamide layer [174]. This approach reduced polymer chain mobility, resulting in a denser, thermally stable membrane. Thermogravimetric analysis showed that adding just 0.0245 wt% TiO_2_ increased the onset of an intense degradation temperature between 530 and 550 °C.

The chemical stability of polymeric membranes can be defined as their ability to withstand different chemical environments, such as bases, acids, oxidizing, or reducing agents, without being chemically degraded. Its chemical stability is an essential determining factor to sustain functioning and preserve its structural integrity for a prolonged period. Resistant to deterioration, chemically stable polymers, such as polyimides, PVDF, PSf, and PTFE, are favored for liquid separation membranes in harsh environments. On the contrary, PVA, PAN, cellulose acetate, and polyethylene are appropriate for not demanding applications, as they can break down in harsh chemical environments, decreasing the membrane’s durability and performance [175]. During the liquid separation procedure, swelling and chemical deterioration may result from the interaction between the membranes with solvents, acids, bases, etc. [176,177].

### 6.2. Fouling and Degradation

During separation processes, particles in the retentate often accumulate on the membrane surface, forming a cake layer that diminishes membrane performance by reducing flux. This issue, known as membrane fouling, is particularly prevalent in liquid separation applications, such as oil–water separation and water desalination. Fouling occurs when suspended impurities, such as iron, aluminum, manganese, clay, silt, organic molecules, and biological materials, including bacteria and biofilms, adhere to the membrane surface, causing blockages [178]. As fouling intensifies, transmembrane pressure rises, necessitating increased energy to sustain the initial flux. Moreover, fouling negatively impacts the membrane’s selectivity and rejection efficiency, with common foulants, including macromolecules, salts, colloids, and emulsions, which can cause blockages [179]. 

Protein adsorption studies are often conducted to assess a membrane’s antifouling properties. Hydrophilic surfaces, which attract water and reduce protein adsorption, effectively prevent fouling. Various strategies, including surface modifications using nanoparticles, copolymers, and hydrophilic monomers, have created fouling-resistant polymeric membranes. Techniques such as coating, UV irradiation, and grafting have been reported [2].

**Figure 19:**
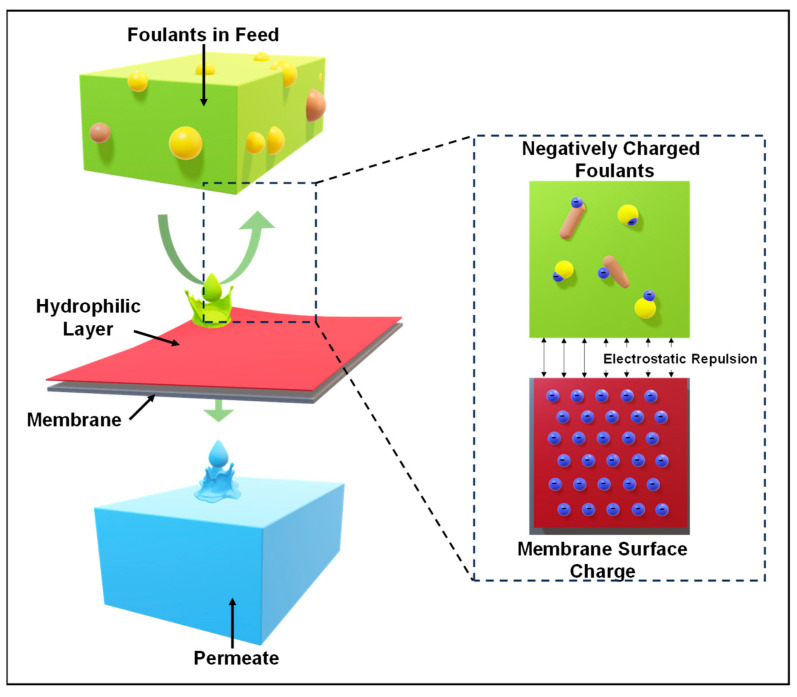
Schematic diagram demonstrating the antifouling mechanism of the membrane.

To mitigate foulant deposition, one effective strategy is the application of antifouling agents to the membrane surface. As depicted in Figure 19, the antifouling mechanism involves applying a thin coating layer directly onto the membrane surface or using cross-linking agents to improve resistance to fouling. For example, Akbari et al. [180] have shown that coating a polyamide nanofiltration membrane with chitosan markedly enhances its antifouling performance and increases pure water flux. Additionally, the antifouling properties of polymeric membranes can be further improved by using various copolymers or by integrating hydrophilic polymers with the base polymeric materials.

Polymeric membranes, including those made from materials such as polyurethane, polyacrylonitrile, polyamide-imide, polyacrylonitrile-g-PEO, cellulose acetate, and poly(vinyl pyrrolidone) composites, have shown promising antifouling capabilities. These properties can be enhanced by modifying the membrane surface with various functional groups, such as amines, amides, carboxylates, sulphones, and epoxides. Techniques such as UV irradiation have effectively improved the antifouling performance [178,181].

The employment of advanced polymeric membranes has resulted in improved antifouling properties and membrane durability, as shown by many studies. The membrane’s antifouling effectiveness also depends on the design of the membrane module. Cleaning the membrane at definite time intervals is also crucial, as it helps maintain the designed membranes’ antifouling behavior [182]. Another important criterion of the membrane that significantly impacts the fouling process is the surface features of the membrane. This suggests that addressing fouling issues in membranes can be achieved by applying antifouling coatings that are carefully engineered to modify the membrane’s chemical and physical intrinsic surface. Adopting innovative strategies and methodologies is essential, given that each type of foulant interacts with the membrane differently [183]. Two main approaches are typically employed to fabricate antifouling membranes: passive and active. The passive antifouling strategy aims to prevent the initial foulants from adhesion to the membrane surface without modifying the intrinsic properties of the foulants.

In contrast, active antifouling involves decomposing or removing the foulants’ chemical structures. These strategies often fail to prevent bacterial and microbial colonization, forming biofilms. Therefore, a more advanced approach is needed to develop antifouling membranes with enhanced properties that can effectively prevent biofilm growth and control microbial colonization [184]. The application of membranes containing nanomaterials, such as nanoparticles, nanowires, and nanosheets, has significantly broadened the potential for developing new types of antifouling membranes. In the future, research and development should prioritize the development of membranes with zero fouling so that they can compete with other technologies and be scaled up for commercial use.

## 7. Emerging Technologies and Trends

Membrane-based methods are generally used in desalination, water reuse, wastewater treatment, and water purification, including removing heavy metals [5,140] and micropollutants [10]. These technologies have been applied individually or in combination with others to advance membrane performance. Recent research has focused on synthesizing a new generation of membranes that offer improved antifouling properties, higher recovery flux, biodegradability, and reduced energy requirements, as outlined below [62].

Antifouling membranes: Recent advancements in antifouling membranes encompass the development of hybrid nanocomposite membranes, the incorporation of organic modifiers, techniques involving IP, and the use of acid monomers. The previous study [140] mentioned that thin-film polyamide membranes are fabricated and embedded with COOH-TiO_2_ nanoparticles, demonstrating enhanced antifouling properties and effective rejection of heavy metal ions using a novel VP-IP approach.

Membrane surface modification: This process can be accomplished through two primary methods: (a) Physical surface modification, which includes the application of surfactants or polyelectrolytes to the membrane surface or coating the membrane with hydrophilic polymers. These methods enhance electrostatic repulsion and help minimize fouling. (b) Chemical modification, which involves incorporating active functional groups into the membrane through chemical reactions to improve its overall performance [185,186].

Fabrication of membranes utilizing green polymers and solvents: The residual waste from making nanoparticles using green methods has been utilized to synthesize a nano-polymer membrane. This advancement is a component of using membrane technology to minimize pollutants in environmental waste and industrial wastewater before reaching water courses [187]. Green solvents have been employed to create polymeric membranes. To promote environmentally friendly advancements in membrane technology, replacing hazardous solvents with suitable green alternatives is essential [188]. A methodology known as solvent-induced polymerization enhances the yield of uniform, crystalline, porous, and structurally robust polymers. This approach presents valuable prospects for water and wastewater treatment [189]. Greener methods are also employed to fabricate TFN membranes [5,62].

Thin-film nanocomposite membrane: The advancement of polymeric nanofiller-based separation membranes continues to evolve, with several promising trends and developments emerging in the field. Various nanoparticles have been included in polymer matrices, including zeolites, graphene oxide (GO), metal oxide, covalent-organic frameworks (COFs), carbon nanotubes (CNT), and metal-organic frameworks (MOFs), to improve the performance of the separation membranes [4,8,10,18]. Depending on their functional groups or nanoscale holes in the membrane, these nanoparticles can enhance the separation efficiency and provide antibacterial and antifouling properties [141]. Incorporating nanoparticles into membranes can improve solvent recovery and desalination, which are just a few energy-efficient separation processes.

Biodegradable membrane: Biodegradable polymers can be derived from plants, animals, and synthetic materials. Biodegradable polymers are often blended with other polymers to enhance their properties and achieve specific performance characteristics [125,126,127].

Aside from these, electrospun aramid nanofiber membranes and membrane bioreactors (MBRs) are also gaining prominence for their exceptional performance in various applications. While PAN-based electrospun nanofiber membranes are employed for advanced separation, MBRs have been utilized to produce renewable energy. Polymeric membranes have unlocked numerous opportunities for applications in separation processes. Thus, the potential for new technologies, trends, and applications for membrane utilization continues to expand.

## 8. Successful Industrial Implementations

Several implementations of membrane-based technology in industries have been reported so far in various applications, including water treatment [190], food and beverages [191], the energy sector [192], gas separation [193], etc. Commercialized membranes are used for water treatment and desalination. Ramdani et al. [190] compared the performance of two commercial TFC NF membranes (NF90 and NF270) regarding fluoride retention, desalination efficiency, and energy consumption. The highest permeate flow rate was found for NF270, while NF90 had the highest retention efficiency. Chandrapala et al. [191] utilized three commercialized NF membranes to separate undissociated lactose from charged lactate anions in an acid whey system. Membranes are also implemented in energy-related applications. A study by Sadrameli et al. [192] utilized membranes having commercialized benefits for the effective purification of biodiesel by removing the impurities present in biodiesel, such as glycerol, diglycerides, and triglycerides. These membranes were utilized on a large scale for the above-mentioned applications [194]. Therefore, due to economic feasibility, easy processing, less wastage, and negligible byproducts, membrane separation technology has emerged as a prominent technology for industrial implementation.

## 9. Future Directions in Polymeric Membrane Research

The research on polymeric membranes is one of the most evolving and dynamic fields, with many potentials in water treatment, water purification, and biomedical devices. The primary objective of membrane research has continuously been to innovate novel membrane materials with enhanced and modified properties compared to pre-existing membrane materials. Future advancements in membrane materials and fabrication will hinge on the sustainability, innovation, and scalability of novel approaches and the stability of resulting membranes. Additionally, delivering rapid prototype feasibility and engaging in interactive processes with risk-taking and visionary industries is crucial.

The advancement of society and the growing global concern over environmental pollution underscore the critical importance of green and sustainable synthesis. However, only a few studies currently adopt green synthesis approaches for preparing polymeric membranes. Furthermore, most currently used green techniques often fail to achieve complete objectives. However, there is ample potential for progress. Therefore, developing new strategies for polymeric membranes is essential to achieve the ultimate goal of green synthesis. To advance the field, future research should prioritize enhancing the synthesis efficiency of eco-friendly solvents, such as tributyl O-acetyl citrate (ATBC), ethyl lactate, ionic liquids (ILs), supercritical CO_2_ (ScCO_2_), γ-butyrolactone (g-BL), tributyl citrate (TBC), triethyl citrate (TEC), dioctyl sebacate (DOS), triacetate ester of glycerol (triacetin), methyl lactate, and propylene carbonate (PC), aiming to achieve or surpass the performance levels of traditional hazardous solvents. Additional investigations into these green solvents’ polarity, viscosity, and cloud point are essential for further development.

Fouling and high energy consumption remain persistent challenges in non-equilibrium pressure-driven processes, highlighting the need for continued research to develop sustainable solutions. These solutions may involve implementing cost-effective yet robust pre-treatment methods or designing membranes that resist fouling. Recent advancements in fabrication technologies, including additive manufacturing (AM), are progressively being investigated for manufacturing membrane modules and desalination cell components with intricate designs [195,196]. These complex structures can mitigate reduced concentration polarization, mass transfer, high fouling, and significant pressure drops. A notable development is 4D printing, an extension of 3D printing, which enables materials to transform their physical properties when exposed to external stimuli. This innovative approach has attracted interest in enhancing the performance of membrane module components. Spiral-wound membrane modules (SWMs) are the most frequently produced using 3D-printing techniques [197].

Emerging modeling techniques, such as molecular dynamics simulation (MDS) and artificial intelligence (AI) tools, including fuzzy logic (FL), genetic algorithms (GA), and artificial neural networks (ANNs), are playing a significant role in advancing sustainability in membrane-based water treatment [198]. Hybridizing AI tools is suggested to achieve more robust global optimization, and expanding their application to include broader water resource management could further enhance their impact [199].

## 10. Conclusions

Polymeric membranes have emerged as pivotal components in modern separation technologies, offering versatile and efficient solutions for liquid separations across various industries. This review has comprehensively examined the fundamental principles, material innovations, fabrication techniques, and performance enhancement strategies that define the current landscape of polymeric membrane technology. Despite significant advancements, several challenges remain, including membrane stability, fouling, scalability, and environmental impact. Addressing these challenges necessitates ongoing research and development, focusing on advancements in material science and membrane design. Integrating novel materials, such as nanocomposites and advanced hybrid structures, holds promise for overcoming these limitations and achieving superior separation performance. The commercialization of polymeric membranes is advancing steadily, with successful implementations in water treatment and beyond. However, the full potential of these technologies is yet to be realized. Future research should focus on developing more sustainable, cost-effective, and high-performing membranes to meet the growing demands of industrial applications. In conclusion, polymeric membranes are set to play an increasingly important role in addressing global challenges related to resource conservation, environmental protection, and industrial efficiency. By continuing to innovate and refine these technologies, we can unlock new possibilities for liquid separations, driving progress in established and emerging fields.

## Figures and Tables

**Table 1:** Some of the high-temperature-resistant polymers [153,154,155].

Abbreviation	Polymer	Tg (°C)	CUT ^a^ (°C)	HDT ^b^ (°C)
PAI	Polyamide-imide	275	250	279
PI	Polyimide	250	288	246
PES	Polyethersulfone	224	177–204	204–238
LCP	Liquid-crystal polymer	_	204–232	321–335
PEI	Polyetherimide	213	177–204	199–216
PSF	Polysulfone	190	149–171	171–182
PEK	Polyetherketone	165	260–288	>316
PEEK	Polyetheretherketone	143	204–232	177–321
PPA	Polyphthalamide	134	204–232	277–285
PPS	Polyphenylene sulfide	92	204–232	149–288
PEKK	Polyetherketoneketone	165	232–260	>316
FEP	Fluorinated ethylene propylene	149		
PFA	Perfluoroalkoxy	_	240–260	_

^a^ Continuous use of temperature. ^b^ Heat deflection temperature.
